# Supplementary material for: Evaluation of noninvasive biospecimens for transcriptome studies
Source: BMC Genomics. 2023 Dec 19;24:790. doi: 10.1186/s12864-023-09875-4 (PMC10729488; doi:10.1186/s12864-023-09875-4)
Supplement: Supplementary file 1 — Additional file 1: Supplementary Figure 1. a. Outcome of library preparation QC per donor, collection, and preparation. The crosses indicate a failed sample, and the numbers correspond to the collection. b. Measured RIN vs computationally-derived transcript integrity number (TIN) per sample. Passing is determined by 1 million protein-coding depth threshold. c. RNA yield distribution per tissue type. Buccal median = 1054.2ng, Q1 = 735.7ng, Q3 = 1292.55ng. Hair median = 1444.1ng, Q1 = 606.2ng, Q3 = 2530.5ng. Saliva median = 4705.4ng, Q1 = 2278.5ng, Q3 = 10920.35ng. Urine median = 253.4ng, Q1 = 98ng, Q3 = 1642.2ng. d. cDNA average size for Loseq and SmartSeq preparations across noninvasive tissues. e. RNA yield per donor and tissue. Each data point is a collection. Buccal Levene P = 0.05, Hair Levene P = 0.01, Saliva Levene P = 0.2, Urine Levene P = 0.0002. f. Total reads sequenced per sample, colored by prep. g. Categorization of gene types for uniquely mapped reads mapping to genes across tissues. Supplementary Figure 2. a-c quality statistics for all samples across all library preparations: a. b. Samples remaining and genes detected depending on depth threshold. Dotted line indicates preparation QC threshold of 1 million. c. Proportion of reads mapping to exonic, intergenic, and intronic genomic features. Pass indicator is based on preparation QC threshold of 1 million. d-f quality statistics for only Loseq samples: d. e. Samples remaining and genes detected depending on depth threshold. Dotted line indicates Loseq QC threshold of 2.5 million. f. Proportion of reads mapping to exonic, intergenic, and intronic genomic features. Pass indicator is based on Loseq QC threshold. Supplementary Figure 3. a. Intersection of genes with greater than zero median expression across library preparations. b. c. Comparison of gene length and GC content of genes uniquely captured in a given preparation. d. Comparison of median gene expression levels of library preparation replicates. [file 12864_2023_9875_MOESM1_ESM.docx]

**Supplementary Figure 1. a.** Outcome of library preparation QC per donor, collection, and preparation. The crosses indicate a failed sample, and the numbers correspond to the collection. **b.** Measured RIN vs computationally-derived transcript integrity number (TIN) per sample. Passing is determined by 1 million protein-coding depth threshold. **c.** RNA yield distribution per tissue type. Buccal median = 1054.2ng, Q1 = 735.7ng, Q3 = 1292.55ng. Hair median = 1444.1ng, Q1 = 606.2ng, Q3 = 2530.5ng. Saliva median = 4705.4ng, Q1 = 2278.5ng, Q3 = 10920.35ng. Urine median = 253.4ng, Q1 = 98ng, Q3 = 1642.2ng. **d.** cDNA average size for Loseq and SmartSeq preparations across noninvasive tissues. **e.** RNA yield per donor and tissue. Each data point is a collection. Buccal Levene P = 0.05, Hair Levene P = 0.01, Saliva Levene P = 0.2, Urine Levene P = 0.0002. **f.** Total reads sequenced per sample, colored by prep. **g.** Categorization of gene types for uniquely mapped reads mapping to genes across tissues.

**Supplementary Figure 2. a-c** quality statistics for all samples across all library preparations: **a. b.** Samples remaining and genes detected depending on depth threshold. Dotted line indicates preparation QC threshold of 1 million. **c.** Proportion of reads mapping to exonic, intergenic, and intronic genomic features. Pass indicator is based on preparation QC threshold of 1 million. **d-f** quality statistics for only Loseq samples: **d. e.** Samples remaining and genes detected depending on depth threshold. Dotted line indicates Loseq QC threshold of 2.5 million. **f.** Proportion of reads mapping to exonic, intergenic, and intronic genomic features. Pass indicator is based on Loseq QC threshold.

e

**Supplementary Figure 3. a.** Intersection of genes with greater than zero median expression across library preparations. **b. c.** Comparison of gene length and GC content of genes uniquely captured in a given preparation. **d.** Comparison of median gene expression levels of library preparation replicates. The median of all replicate 1 samples (15) is compared against the median of all replicate 2 samples (15) per library preparation. The spearman correlation between these is shown. **e.** Comparison of the number of genes detected per tissue and library preparation depending on the RNA yield of the sample. Pearson correlation and p-value is shown.

**Supplementary Figure 4. a. b.** Principal Component Analysis of all samples passing preparation QC thresholds. **c.** Percent variance explained per PC. **d.** ANOVA results for PC ~ tissue and PC ~ preparation. P-values are Bonferroni-corrected for the number of PCs tested (10).

e

**Supplementary Figure 5. a.** Schematic of pipeline for assigning unmapped reads. Briefly, unmapped reads were remapped using decontaminer, normalized using a procedure akin to TPM normalization, then species of interest were filtered based on their abundance and replication across technical replicates. Remaining unmapped reads were investigated using FastQC. **b.** Total number of reads assigned to each category. Mapped = aligned to hg38. Remapped = aligned to microbial species using Decontaminer. Repeated = highly abundant reads identified by FastQC. Unknown = reads not mapped, remapped, or highly abundant. **c.** For each species included in the final analysis, spearman rank correlation between technical replicates is shown with the dots. Bar plot of species abundance with error bars is shown in the background for direct comparison (y-scale of abundance in Figure 2b). **d.** Breakdown of repeated sequence sharing across tissues and library preparations. **e.** Mapping rate per tissue and library preparation as a function of RNA yield. Pearson correlation and p-value is shown.

**Supplementary Figure 6. a.** Variance in gene expression across tissues explained by technical and biological variables. Canonical correlation analysis shows correlation between variables used. **b. c.** Variance in gene expression within each tissue explained by technical and biological variables. Canonical correlation analysis shows correlation between variables used. Only Loseq samples were included in these analyses.

**c**

**Supplementary Figure 7. A.** GEDIT cell type proportion estimates per collection and donor. Top 25% most abundant, condensed cell type categories are shown. **b.** Breakdown of all cell types included in the GEDIT reference. Binning into larger cell type categories is shown. **c.** Cell type proportion estimates looking across samples ordered from lowest to highest RNA yield.

**Supplementary Figure 8. a.** xCell enrichment scores across the noninvasive and select GTEx tissues for cell types corresponding to the GEDIT collapsed cell type categories. Note that the enrichment score does not correspond to a proportion. **b.** Comparison of xCell enrichment scores and GEDIT proportions for cell types shared by both references. Only noninvasive tissues are shown.

**Supplementary Figure 9. a. b. c.** Calculated pi1 versus the null pi1 distribution for every tissue in GTEx. Pi1 was calculated by selecting for significant variants (q-value <=0.05) with MAF > 0.05 and minimum effect size greater than the maximum minimum across GTEx tissues (kidney cortex 0.32) that were present in the noninvasive dataset. The null distribution was generated by performing 1000 samples of size equivalent to the number of overlapping gene-variant pairs used for the pi1 calculation for that tissue. **d. e. f.** Histograms of Loseq p-values for gene-variant pairs included in the pi1 calculation.

**Supplementary Figure 10. a.** Number of sex-based upregulated genes per noninvasive tissue type. **b.** Per tissue overlap between significant DE genes in the noninvasive dataset with genes previously found to be significant in the GTEx dataset. **c.** Sex-based differential expression for buccal samples, with no significant genes. FGSEA shows some rank-based gene category enrichment for females.

**Supplementary Figure 11. a.** Ratio of (reference allele count)/(total count) for all heterozygous sites per donor and tissue. **b.** Reference ratio breakdown per VEP annotation.

**Supplementary Figure 12. a.** Total number of disease-relevant genes per OpenTargets ontology category. Genes with >= 5 separate sources of evidence were included in the final analysis. **b.** Number of genes per tissue following minimum expression level thresholding and overlap with the HPA tissue-elevated gene list. The top 3,411 most expressed genes per tissue were included in the analysis. **c.** Summed evidence scores (SESs) for all GTEx and noninvasive tissues.

**Supplementary Figure 13. a.** Proportion and total OMIM gene capture per noninvasive tissue type, depending on minimum median TPM threshold. **b.** Proportion of OMIM gene capture per collection, donor, and tissue using a minimum expression threshold of 0.1 TPMs. **c.** Clustering of noninvasive and select GTEx tissues based on median OMIM gene expression.

**Supplementary Table 1.** Breakdown of reaction cost (as of August 2022) per reagent using our in-house method (Loseq) versus TruSeq Stranded mRNA Library Prep and Takara SMART-seq V4 commercial kits. The cost of Ampure XP beads (cat# A63881) is excluded, but it is notably lower per reaction for Loseq and SMART-seq preparations due to smaller volume requirements.

| Catalog Number | Reagent Name | Cost per reaction |  |
| --- | --- | --- | --- |
| Loseq | | | |
| 50-196-5299 | KAPA HiFi HS reaction mix | $1.38 |  |
| 30281-2 | NxGen® RNAse Inhibitor | $0.69 |  |
| EP0752 | Maxima H Minus RT | $0.48 |  |
| R0192 | dNTP set (10mM each) | $0.14 |  |
|  | 3' RT Primer (anchor) HPLC purified | $0.28 |  |
|  | drop-TSO HPLC Purification | $0.09 |  |
|  | drop-PCR HPLC Purification | $0.04 |  |
| 20027213 | Nextera UD indexes | $1.75 |  |
| FC-131-1096 | Nextera XT DNA Library Preparation Kit | $8.95 |  |
| 61012 | Dynabeads mRNA Direct Purification kit | $3.75 |  |
| AM8170G | DNase I Buffer (10x) | $0.12 |  |
| AM2224 | DNase I (RNase-free) | $0.03 |  |
| Illumina | | | |
| 20020595 | TruSeq Stranded mRNA | $96.46 |  |
| 20020591 | TruSeq UD indexes | $5.71 |  |
| SMART-seq | | | |
| 634891 | TakaraBio SMART-seq V4 | $55.92 |  |

**Supplementary Table 2.** Comparison of total cost per reaction by library preparation. Loseq percent cost reduction calculated as: (1-(Loseq/Commercial kit))*100

| Library Preparation | Total Cost per Reaction | Loseq Cost Reduction |
| --- | --- | --- |
| Loseq | $17.70 |  |
| Illumina | $102.17 | 82.68% (-$84.47) |
| SMART-seq | $55.92 | 68.35% (-$38.22) |
